# Fully automated preoperative liver volumetry incorporating the anatomical location of the central hepatic vein

**DOI:** 10.1038/s41598-022-20778-4

**Published:** 2022-10-01

**Authors:** Sven Koitka, Phillip Gudlin, Jens M. Theysohn, Arzu Oezcelik, Dieter P. Hoyer, Murat Dayangac, René Hosch, Johannes Haubold, Nils Flaschel, Felix Nensa, Eugen Malamutmann

**Affiliations:** 1 Institute of Diagnostic and Interventional Radiology and Neuroradiology, University Hospital Essen, Essen, Germany; 2 Institute of Artificial Intelligence in Medicine, University Hospital Essen, Essen, Germany; 3 Department of General, Visceral and Transplantation Surgery, University Hospital Essen, Essen, Germany; 4 Department of Surgery, Medipol University Hospital, Istanbul, Turkey

**Keywords:** Three-dimensional imaging, Computed tomography, Liver

## Abstract

The precise preoperative calculation of functional liver volumes is essential prior major liver resections, as well as for the evaluation of a suitable donor for living donor liver transplantation. The aim of this study was to develop a fully automated, reproducible, and quantitative 3D volumetry of the liver from standard CT examinations of the abdomen as part of routine clinical imaging. Therefore, an in-house dataset of 100 venous phase CT examinations for training and 30 venous phase ex-house CT examinations with a slice thickness of 5 mm for testing and validating were fully annotated with right and left liver lobe. Multi-Resolution U-Net 3D neural networks were employed for segmenting these liver regions. The Sørensen-Dice coefficient was greater than 0.9726 ± 0.0058, 0.9639 ± 0.0088, and 0.9223 ± 0.0187 and a mean volume difference of 32.12 ± 19.40 ml, 22.68 ± 21.67 ml, and 9.44 ± 27.08 ml compared to the standard of reference (SoR) liver, right lobe, and left lobe annotation was achieved. Our results show that fully automated 3D volumetry of the liver on routine CT imaging can provide reproducible, quantitative, fast and accurate results without needing any examiner in the preoperative work-up for hepatobiliary surgery and especially for living donor liver transplantation.

## Introduction

Accurate assessment of the liver volume is essential in the preoperative work-up for hepatobiliary surgery. Future remnant liver volume is a key factor for oncologic liver resections, as well as for optimal donor selection in living donor liver transplantation. The postoperative morbidity and mortality rate of living donor liver transplantation correlates significantly with functional liver volume for the recipient and the donor. The total liver volume has a known relation to the body weight, which is described to be around 2.5% in healthy subjects^1^. In order to avoid liver dysfunction in the recipient, the graft weight to body weight ratio should be at least 1%. For non cirrhotic livers in healthy subjects, resections of up to 80% of the liver volume can be tolerated. The remnant volume in living donors should be at least 30% of the liver volume since donor safety has absolute priority. The generally accepted tool for the estimation of the liver volume and its lobes is the contrast enhanced computed tomography or magnetic resonance imaging. The volumetry of the right and left liver lobes is extremely time-consuming and, depending on the experience of the examiner, afflicted with an estimation error of 5%-35%^2–4^. There are several new software tools for automated and accurate volumetry developed in the last few years. These tools are still very time-consuming and depend on the experience of the examiner. The aim of our study was to develop a convolutional neural network (CNN) based tool for fully automated preoperative assessment of right and left liver volumes with respect to the central hepatic vein from standard computed tomography scans of the liver, without the need of an experienced examiner, which is reproducible and fast.

## Materials and methods

### Ethics statement

The study was approved by the Institutional Review Board of the University Hospital Essen (approval number: 19–8804-BO). Written informed consent was waived by the ethics board due to the retrospective nature of the study. All methods and procedures were performed in accordance with the relevant guidelines and regulations.

### Dataset

In this work, a dataset consisting of 100 abdominal/liver CT scans (53 female, 47 male) with a slice thickness of 5 mm was collected at the University Medicine Essen. For validation an external dataset consisting of 30 CT scans (12 female, 18 male) with a slice thickness of 1.5 mm was collected from the Medipol University Hospital in Istanbul, Turkey. The validation data was resampled to 5 mm slice thickness for a unified voxel spacing. All CT scans were performed with multidetector-row CT systems, mainly with 16- or higher detector-row systems. Venous phase imaging was performed 70–80 s after intravenous administration of a contrast agent, with a median tube voltage of 100 kVp ranging from 90 to 120 kVp. Please refer to the supplement for detailed information about the scanner parameters. The training data set was annotated by a single reader with right and left lobe segments, whereas the test data set was annotated by three different readers. In addition, a standard of reference (SoR) was derived from all three readers by majority voting. Voxels without majority annotation were marked with an ignore label and thus ignored in subsequent analyses.

### Network design

A popular network architecture choice for medical image segmentation is the U-Net^5^, especially promoted to be good for optimization with very few examples. Shortly after, a modified version for 3D imaging, namely U-Net3D^6^, was presented, in order to utilize the spatial context of volumetric data. However, U-Net and their simple variants suffer from some basic problems regarding the feature processing flow and therefore most recent formulations of those networks use more complex skip connections instead of simple identity skip connections between the encoder and decoder.

**Figure 1:**
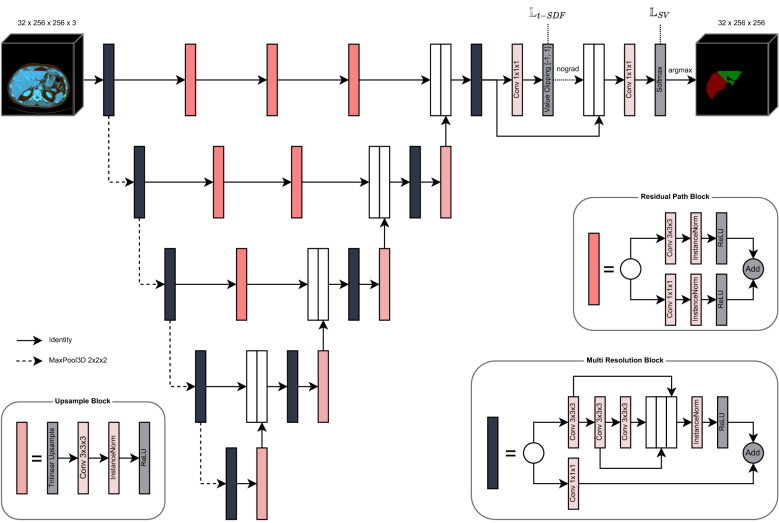
Architecture of the Multi-Resolution U-Net for liver lobe classification with auxiliary truncated Signed Distance Field (t-SDF) regression.

In this paper, we adopted the architecture of the Multi-Resolution U-Net^7^ from 2D to 3D imaging by replacing all 2D convolutions and max-pooling layers with their respective 3D counterparts. Additionally, since GPU memory is the main limitation when working with 3D data, batch normalization^8^ layers were replaced by instance normalization^9^ layers in order to be able to use batches with only a single example. The complete architecture as well as all building blocks and utilized layers are visualized in Fig. 1.

In short, the network architecture involves two changes to the standard U-Net architecture. First, instead of two successive convolutional layers with normalization and activation like in VGG networks^10^, multiple receptive fields are computed by factorizing a 7 × 7 × 7 convolution into three successive 3 × 3 × 3 convolutions and concatenating the intermediate results along the channels axis, followed by a normalization and activation layer. In addition to the factorized convolution, a simple 1 × 1 × 1 convolution is used as a residual path and both results are added as in residual networks (Res-Nets)^11^. The second modification concerns the skip connections between the encoder and decoder. Instead of a simple identity function, multiple residual blocks are used in order to close the so-called “semantic gap”.

For the final classification layer, the softmax activation function was chosen. Additionally, we introduced an auxiliary classifier to solve a truncated Signed Distance Field (t-SDF) regression task ^12^, which will be explained in more detail in the next section. The final loss function for optimization consists of a weighted combination of a categorical cross entropy loss and generalized soft dice loss^13,14^, similar to Isensee et al. ^13^, as well as a L1 loss for the t-SDF regression.

### Preprocessing

**Figure 2:**
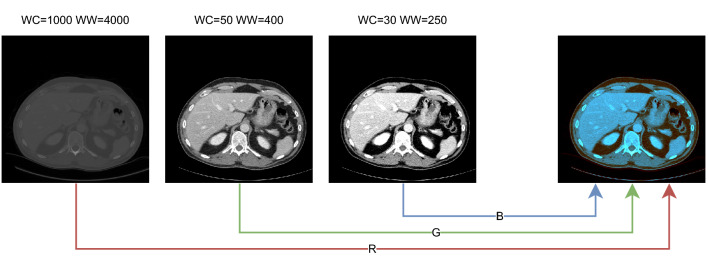
Visualization of the network inputs with multiple hounsfield windows applied. WC = Window Center, WW = Window Width. From left to right: all Hounsfield Units within 12-bit scanner data, abdominal soft tissue window, liver tissue window, and the composite RGB image.

All CT images have a variable number of slices and were resampled to an axial resolution of 256 × 256 in order to reduce the computational load and amount of required GPU memory. CT images are by default stored as Hounsfield Units (HU), a standardized measurement of voxel density. For model input, the HU values were rescaled and clipped to [− 1, 1] using three different HU windows. A sample composite RGB image is shown in Fig. 2. Theoretically, a single HU window including all available information should be sufficient. However, it was empirically observed that using multiple windows led to better convergence, especially with mixed precision training.

During training, the CT images were further processed by employing a random data augmentation pipeline. Since the dataset at hand is rather small, augmentations can help to train better generalizing networks. First, random rotations within − 10° and 10° were applied, which mimics minor rotary patient movements. Second, axis independent scale augmentations in the range of 80–120% were applied. Third, the resampled CT images were randomly cropped to 32 × 128 × 128 voxels. This can be interpreted as loss sampling and also forces the network to learn more spatially-aware features.

**Figure 3:**
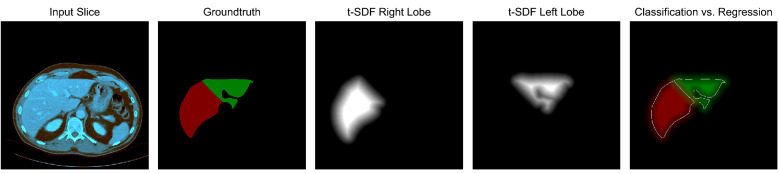
A multi-class segmentation converted to a truncated Signed Distance Field (t-SDF) for an auxiliary regression task. The right hand image shows the difference between the classification and regression boundary. For t-SDF regression, the network is forced to learn more about the spatial context.

For the t-SDF regression, the groundtruth multi-class labels need to be converted into distance maps. A fast Python implementation of the euclidean distance transform (EDT) can be found at https://github.com/seung-lab/euclidean-distance-transform-3d, which is even compatible with 3D data, multi-label annotations, and anisotropic voxel spacing. As visualized in Fig. 3, the signed EDTs of both class labels are computed and in the last column, the difference between hard classification boundaries and t-SDF regression is visualized. Normally, an EDT only computes the distance inside a label to the closest border pixel. Using the above-mentioned library for multi-label distance computation, the binary class label mask is shifted up by one, so that the background pixels have value of 1 and foreground pixels have value 2. The signed EDT can then be efficiently computed using the masking operation for regions with value 1 and 2. Distances are by default unbounded, but unbounded value regression does not work well with the limited receptive field of CNNs. Hence, the distances are clipped to [− 25, 25] mm to the closest border pixel and afterwards rescaled to [− 1, 1].

### Optimization

As an optimizer, Adam^15^ with a constant learning rate of 0.0001 and decoupled weight decay regularization^16^ with 0.0001 was chosen. Model training was performed using a fivefold cross-validation scheme, thus yielding five estimates for each model configuration and the support for late fusion model ensemble. The networks were trained with a batch size of 1 for 500 epochs each (or 40.000 steps) and were evaluated every 10 epochs (or 800 steps) to monitor the Sørensen-Dice coefficients on the respective validation splits. Model weights were saved if a better average Sørensen-Dice coefficient was found on the respective cross-validation fold.

As mentioned before, the CT images have a variable number of slices and during training a crop of 32 × 128 × 128 voxels is sampled. However, for computing the complete segmentation on an variable-sized abdominal CT scan a sliding window approach is utilized. The sliding window approach samples crops of 32 × 256 × 256 voxels with 75% overlap along the z-axis. In order to stabilize the predictions at the edges of the crops, a weighting scheme is used for aggregation of the probability maps. Thus, full weight is given to the central 16 slices and an interpolated weight to nearly zero for 8 slices on each side.

## Results

Evaluation was performed using commonly used metrics for medical semantic segmentation tasks: Sørensen-Dice coefficient, precision, recall, relative volume difference (RVD), and volume difference (VD). First, the Sørensen-Dice coefficient, precision, and recall measure the overlap between the manual and automatic liver/lobe segmentations. Second, the VD metric measures the difference in milliliter (ml) and RVD additionally normalizes the difference by the manually measured volume, which are more relevant metrics for clinical treatment.

**Figure 4:**
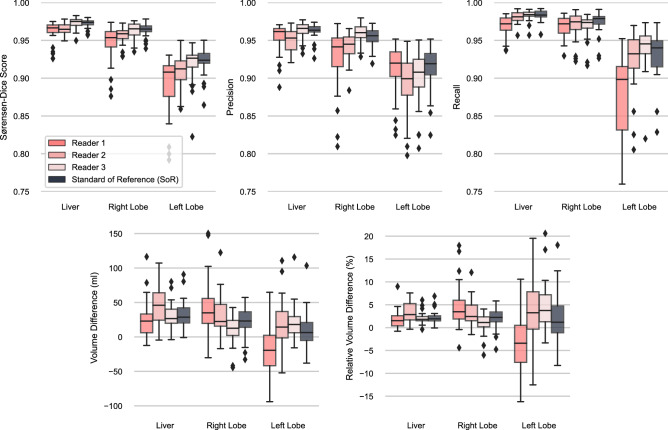
Evaluation of the trained model ensemble on the test dataset. Each CT scan was annotated by three different readers and additionally a standard of reference was created by majority voting.

In Fig. 4, the model performance is evaluated on the test set and compared between all three readers as well as the derived standard of reference. Model performance is very high with a Sørensen-Dice coefficient of 0.9726 ± 0.0058, precision of 0.9623 ± 0.0101, recall of 0.9832 ± 0.0068, RVD of 2.19 ± 1.40%, and a VD of 32.12 ± 19.40 ml compared to the SoR liver annotation. For the SoR right lobe and left lobe annotation, Sørensen-Dice coefficient is 0.9639 ± 0.0088 and 0.9223 ± 0.0187, precision is 0.9537 ± 0.0128 and 0.9141 ± 0.0290, recall is 0.9747 ± 0.0150 and 0.9319 ± 0.0311, RVD is 2.22 ± 2.30% and 2.07 ± 5.52%, and the VD is 22.68 ± 21.67 ml and 9.44 ± 27.08 ml, respectively. Between annotators there is a clear variability visible, the largest deviations can be found in the annotation of the left lobe.

**Figure 5:**
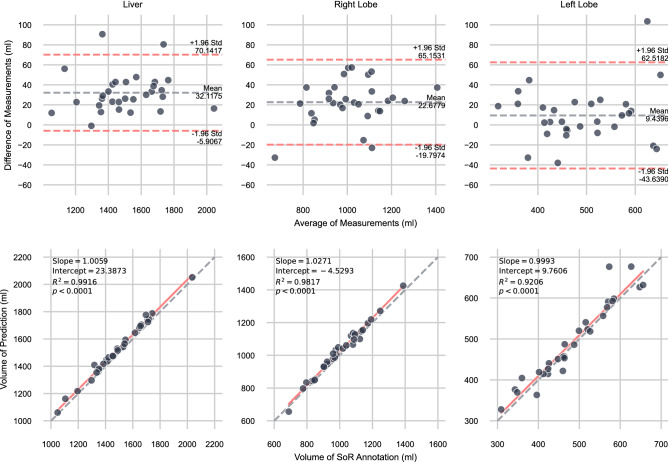
Analysis of the predicted volume versus the volume of the SoR groundtruth. (top) Bland–Altman diagrams (bottom) Scatter plots with OLS regression analysis.

In Fig. 5, the VD between the model prediction and SoR annotation is further analysed using a Bland–Altman diagram and scatter plots of the volumes including ordinary least squares (OLS) regression analysis. All three OLS regression models were verified with the F-Test (*P* < 0.0001) and the respective variables were tested on normal distribution using the Shapiro–Wilk test. The tool has a bias and over-predicts for all three evaluated labels with an acceptable margin. However, since the CT scans have a slice thickness of 5 mm, it is difficult for a human reader to annotate the correct decision boundary if it is somewhere in between two slices. After manual review of the model predictions, most of the segmentation differences come from mismatches between adjacent slices near the heart and less from in-plane inaccuracies.

**Figure 6:**
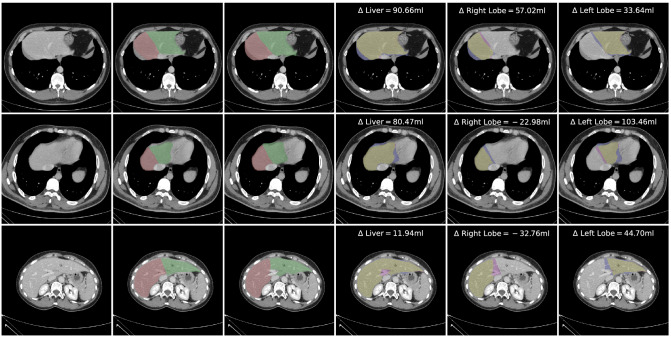
Case studies of three outliers identified by the bland–altman plot. From left to right: original image, SoR annotation, prediction, and error maps for liver, right lobe and left lobe. Error maps show true positives in yellow, false positives in blue, false negatives in pink, and ignored voxels in cyan.

For further inspection of the model errors, three outlier cases were identified by Fig. 5. Those cases were analysed and visualized in Fig. 6. In the top row, the ensemble over-segmented the right lobe and includes the diaphragm. In the middle, the left lobe is over-segmented into regions of the heart, however, due to partial volume effects the real decision boundary is partially unclear. Again, in this case the diaphragm is partially included in the right lobe as well. In the bottom row, the portal vein is excluded on multiple slices from the overall liver prediction. Additionally, the separation line between the right and left lobe is shifted and under-segments the right lobe.

In addition, the annotations of all three readers and the prediction of the proposed tool were evaluated against each other in Table 1. The table shows the Sørensen-Dice coefficients in the upper triangular matrix and the R^2^ coefficients of an OLS regression analysis in the lower triangular matrix. Strong correlation can be observed for all three labels, however, there are clear differences between individual readers as well as readers to the predicted volumes.

**Table 1:** Evaluation between the three readers (R1-R3) and the proposed tool (AI). The upper triangular matrix states Sørensen-Dice coefficients. The lower triangular matrix states R2 coefficients of the respective volumes.

	R1	R2	R3	AI
**Liver**				
R1		0.9616 ± 0.0156	0.9636 ± 0.0152	0.9613 ± 0.0144
R2	0.9771		0.9771 ± 0.0113	0.9652 ± 0.0072
R3	0.9915	0.9797		0.9731 ± 0.0069
AI	0.9838	0.9798	0.9936	
**Right lobe**				
R1		0.9478 ± 0.0255	0.9479 ± 0.0260	0.9457 ± 0.0245
R2	0.9252		0.9675 ± 0.0123	0.9551 ± 0.0119
R3	0.9481	0.9704		0.9634 ± 0.0104
AI	0.9375	0.9622	0.9846	
**Left lobe**				
R1		0.9028 ± 0.0472	0.8991 ± 0.0463	0.8931 ± 0.0380
R2	0.8870		0.9338 ± 0.0266	0.9085 ± 0.0231
R3	0.8969	0.8868		0.9191 ± 0.0247
AI	0.8865	0.8590	0.9330	

## Discussion

In this study, we developed and evaluated a CNN-based tool for fully automated liver volumetry on preoperative CT scans considering the anatomical location of the central hepatic vein. This fully automated tool is reproducible, quantitative, fast and accurate and does not need an examiner, which means it is not dependent on the experience of the examiner. The separate volumetry of the right and left lobe is necessary for major oncologic liver resections and is absolutely essential for living donor liver transplantation.

The developed tool achieves Sørensen-Dice coefficients of 0.9726 ± 0.0058, 0.9639 ± 0.0088, and 0.9223 ± 0.0187 for the whole liver, right lobe, and left lobe, respectively. Moreover, linear regression analyses show statistically significant correlations of the predicted volumes compared to the SoR annotation volumes with R^2^ coefficients of 0.9916, 0.9817, and 0.9206.

The idea of automated volumetry, which is fast and especially examiner independent, is not new. There are several studies with the same goal.

The importance of an accurate liver volume assessment preoperatively for living donor liver transplantation is described by Goja et al. using their study cohort of 842 donors. The estimated graft weight was compared to the actual graft weight and overestimation of the left lobe weight and underestimation of the left lateral lobe was detected, which can mislead surgeons in their decision on living donation^17^.

Meyer et al. calculated in their study the remnant liver volume using semi-automated volumetry software. There were 24 patients included in the study, scheduled for hemihepatectomy due to histologically proven primary or secondary hepatic malignancies. The intraoperative weights of the resected hemihepatectomy specimens were determined in the operating room and a conversion factor was determined based on that calculation. It has been shown that the preoperative prediction was more accurate using semi automated software^18^. It is difficult to draw statistically significant conclusions with the relatively low number of 24 patients. However, due to the semi automated approach an experienced radiologist is still needed for preoperative volumetry. The time needed for the semi automated volumetry was not described in the study. In contrast, our study presents a fully automated tool to assess the right and left liver volume based on routine contrast enhanced CT-scans without the need of any annotation.

Another semi automated software to calculate liver volume is presented by Bozkurt et al. The right/left liver volumes of 41 living liver donors were calculated by the surgeon using the semi automated software tool. The same CT-scans of the donors were used for the manual volumetry of the right/left liver volumes done by an experienced radiologist. The results of the study demonstrated that there is no difference between the two methods of predicting liver volume. Both methods correlate with the intraoperative weight/volume of the explanted liver lobe. The conclusion of the study group was that the preoperative volumetry can be performed also by the transplant surgeons, using the software tool^19^. It is possible that the semi-automatic method is faster than the manual method. However, the time required for the methods was not described in the study. Although this is essential for choosing the best method in everyday business. However, the fully automated tool presented in our study requires only seconds to generate a quantitative volume for the right and left lobes of the liver.

CNN based liver segmentation with manual correction was described by Chelbus et al. MRI scans of 83 patients with primary liver cancer or liver metastases scheduled for selective internal radiation therapy (SIRT) were used for the study, 62 for training and 21 for evaluation. The automated liver segmentation was corrected manually, with a mean Sørensen-Dice coefficient of 0.95. The mean interaction time was 2 min^20^. The main difference to our study is that we were able to generate fully automated volumetry of the right and left liver lobe separately, which is extremely dependent on the experience of the examiner, if performed manually.

Excellent results for fully automated liver segmentation are shown in the study of Winkler et al. The automated assessment of the volumetric analysis was accurate, robust and fast^21^. This study also contained the volumetric analysis of the whole liver. The separate volumetric analysis of the right and left liver lobe is of greater interest in clinical use. The manual assessment requires experience. Consequently, fully automated, accurate and reproducible volumetry of the right and left liver lobe separately improves the results of the volumetry analyses and saves time in clinical practice.

Another study proposed an automatic approach using a convolutional long short-term memory based U-Net for volumetric segmentation of the left lobe, right lobe, caudate lobe, and whole liver on abdominal CTs^22^. The authors show a strong correlation between the manual annotations and the predictions of the tool on 28 patients from a single-center cohort. However, our tool achieves an overall better Sørensen-Dice coefficient for right lobe, left lobe, and whole liver. Small differences due to the additional caudate lobe label in their work might affect the interpretation of the left lobe coefficients. In addition, our automatic tool shows a better correlation with the manual annotations on a multi-center cohort, having less bias and with near identity slope.

In Park et al. a DeepLabV3 + network was employed using 2.5d input images to perform automatic liver and spleen segmentation^23^. The tool achieves a Sørensen-Dice coefficient of 0.973 for the whole liver, which is comparable to the performance in this study. However, the separation between the right and left lobe was performed manually by a reader. Since the correct separation between the right and left lobe is labor-intensive and prone to inter-rater variability, a full automation of the volumetry process is preferable. The authors further correlated the semi-automatically derived volumes of 201 patients with actual graft weights and derived a conversion formula for pre-operative volumetric estimation of graft weight. An evaluation of this conversion formula was conducted on another 374 patients, which resulted in a cross-correlation coefficient of 0.834.

The main shortcoming of our study is that we have not correlated the automated assessed liver volume with the actual graft weight. However the main focus of the study was to develop fully automated volumetric analyses of the right and left liver lobe which showed comparable results to the manual volumetry. The correlation with the actual graft weight in a prospective study will be the next step. Further, the tool could be extended to support right and left lobe donors. This would require small adjustments to the decision boundary along the central hepatic vein. Complementary to this would be the left lateral living donor liver transplantation, where an additional separation between segment I + IVa + IVb and segment II + III would be required.

In conclusion, our study demonstrates that fully automated 3D volumetry of the right and left liver lobe on routine CT imaging provides methodologically and technically reproducible, fast and accurate results without the need for human operators in the preoperative work-up for hepatobiliary surgery.

## Supplementary Information

Supplementary Information.

## Data Availability

The datasets generated during and/or analysed during the current study are not publicly available due to data privacy and legal restrictions, but are available from the corresponding author on reasonable request.

## Abbreviations

2D: Two-dimensional
3D: Three-dimensional
CNN: Convolutional neural network
CT: Computer tomography
EDT: Euclidean distance transform
GPU: Graphics processing unit
OLS: Ordinary least squares
RVD: Relative volume difference
SoR: Standard of reference
t-SDF: Truncated signed distance field
VD: Volume difference
