# Supplementary material for: Fully automated preoperative liver volumetry incorporating the anatomical location of the central hepatic vein
Source: Sci Rep. 2022 Oct 1;12:16479. doi: 10.1038/s41598-022-20778-4 (PMC9526715; doi:10.1038/s41598-022-20778-4)
Supplement: Supplementary file 1 — Supplementary Information. [file 41598_2022_20778_MOESM1_ESM.docx]

# Supplementary File

|  | **Training** | **Validation** |
| --- | --- | --- |
| Convolutional Kernel | B30f(27), Br40d3(6), I30f2(62), I31f2(5) | B(30) |
| CT Model |  |  |
| - SIEMENS Definition | 18 |  |
| - SIEMENS Sensation 16 | 2 |  |
| - SIEMENS SOMATOM Definition | 7 |  |
| - SIEMENS SOMATOM Definition AS+ | 5 |  |
| - SIEMENS SOMATOM Force | 6 |  |
| - SIEMENS SOMATOM Definition Flash | 62 |  |
| - Philips iCT 256 |  | 30 |
| CTDI Phantom Type | IEC Body Dosimetry Phantom | N/A |
| CTDI Volume (mGy) | 7.2±3.0 | 10.3±3.4 |
| Data Collection Diameter (mm) | 500 | 500 |
| Exposure (mAs) | 151.9±49.1 | 151.8±49.7 |
| Exposure Modulation Type | XYZ_EC | Z-DOM |
| Exposure Time (ms) | 500 | 832 |
| Reconstruction Diameter (mm) | 394.5±47.0 | 367.6±25.9 |
| Revolution Time (ms) | 755.8±124.4 | 831.9±1.9 |
| Single Collimation Width (mm) | 0.6(96) | N/A |
| Slice Thickness (mm) | 5.0 | 1.5 |
| Spiral Pitch Factor | 0.6(73), 0.9(23) | N/A |
| Total Collimation Width (mm) | 19.2(23), 38.4(67), 57.6(6) | N/A |
| Tube Current (mA) | 206.1±75.1 | 182.4±59.7 |
| Tube Voltage (kVp) | 90(2), 100(71), 120(27) | 120(30) |
